# Characterization and immunomodulatory activity of exopolysaccharides produced by *Lacticaseibacillus casei* strains on macrophage cultures

**DOI:** 10.1007/s10068-025-02010-x

**Published:** 2025-10-12

**Authors:** Windy Seftiarini, Rarastoeti Pratiwi, Abdul Rahman Siregar, Thu-Ha Nguyen, Widodo Widodo

**Affiliations:** 1 Graduate School of Biotechnology, Universitas Gadjah Mada, Yogyakarta, 55281 Indonesia; 2 Biochemistry Laboratory, Faculty of Biology, Universitas Gadjah Mada, Jl. Teknika Selatan, Sekip Utara, Yogyakarta, 55281 Indonesia; 3 Microbiology Laboratory, Faculty of Biology, Universitas Gadjah Mada, Jl. Teknika Selatan, Sekip Utara, Yogyakarta, 55281 Indonesia; 4 Food Biotechnology Lab, Department of Biotechnology and Food Sciences, BOKU Vienna – University of Natural Resources and Life Sciences, 1190 Vienna, Austria; 5 Department of Animal Product Technology, Faculty of Animal Science, Universitas Gadjah Mada, Jl. Fauna 3 Bulaksumur, Yogyakarta, 55281 Indonesia

**Keywords:** Exopolysaccharide, *Lacticaseibacillus casei*, Probiotics, Immunomodulatory activity, Macrophage cultures

## Abstract

This study aimed to characterize and assess the immunomodulatory capability of exopolysaccharides (EPSs) isolated from *Lacticaseibacillus casei* AP and AG. In a batch fermentation model, *L. casei* AP produced 48 mg/L EPSs, whereas *L. casei* AG produced 62 mg/L EPSs. The percentages of total carbohydrates in EPSs from *L. casei* AP and *L. casei* AG were 79.40 and 81.17%, respectively. Monosaccharide composition analysis revealed the presence of ribose and glucose in EPSs produced by both strains. Fourier transform infrared (FTIR) spectroscopy confirmed the presence of hydroxyl, carboxyl, and amide groups. EPS microstructures showed coarse surfaces, with the main constituents being C, N, O, and Ca. In addition, P was present in the EPSs produced by *L. casei* AP but absent in the EPSs produced by *L. casei* AG. After grown on standard MRS medium, the EPSs isolated from *L. casei* AP and *L. casei* AG exhibited different immunomodulatory activities. These EPSs effectively increased the phagocytic and nitride oxide production of RAW 264.7 cells without toxic effects at concentrations between 50 and 800 μg/mL, suggesting that these strains are interesting producers of EPSs, which could be used in various potential applications in the food and pharmaceutical industries.

## Introduction

Exopolysaccharides (EPSs) are extracellular macromolecules that can be synthesized by various microorganisms. Lactic acid bacteria (LAB) are a group of bacteria that synthesize EPSs (Bouzaiene et al., 2024; Jia et al., 2022; Liu et al., 2023; Van Calsteren et al., 2024; Zhang et al., 2023). EPSs derived from LAB have been reported to be essential components of cosmetic, pharmaceutical, and food products (Jurášková et al., 2022; Nguyen et al., 2020). EPSs have broad application prospects because of their many biological activities, including intestinal microbiota regulation, immune regulation, antioxidation, and antitumour properties (Liang et al., 2024). Therefore, EPSs produced by LAB are considered to significantly benefit human health. Previous studies have demonstrated the immunomodulatory effects of EPSs produced by LAB (Kwon et al., 2020; Lee and Park, 2024; Lee et al., 2024; Woo et al., 2024). Other reports have shown that LAB-derived EPSs enhance specific humoral and cellular immune responses against antigens by increasing the phagocytic activity of macrophages and T/B-lymphocyte proliferation, which in turn promotes the generation of nitric oxide (NO) and cytokines (You et al., 2020). The immunomodulatory effects of EPSs are influenced by their physicochemical characteristics, including their molecular weights and monosaccharide compositions (Hidalgo-Cantabrana et al., 2012; Xu et al., 2019).

Probiotics are live microorganisms that confer health benefits to the host when they are added in sufficient quantities (Hill et al., 2014). *Lacticaseibacillus casei* AP and *L. casei* AG were originally isolated from the gastrointestinal tracts of Indonesian infants less than one month old who consumed breast milk as their sole source of nutrition (Widodo et al., 2012). Previous studies have shown that *L. casei* AP and *L. casei* AG are probiotic bacteria and can be used as starter cultures for milk fermentation (Widodo et al., 2017). The use of these probiotic strains in fermented milk products has been proven beneficial for antihypercholesterolemic effects in mice and improving the fat profile in obese individuals (Widodo et al., 2019, 2021). Milk fermented with *L. casei* AP and *L. casei* AG contains EPSs, which have been reported to affect the physiochemical quality of the final product (Maajid et al., 2022). However, the details of the characteristics and immunomodulatory activity of the EPSs produced by *L. casei* AP and *L. casei* AG have not yet been investigated. In this study, we describe the isolation and characterization of the EPSs produced by *L. casei* AP and *L. casei* AG. Their immunomodulatory activity in the murine macrophage line RAW 264.7 was also evaluated. Our work highlights the unique characteristics of EPSs obtained from human-origin strains, *L. casei* AP and *L. casei* AG, which previously demonstrated health benefit functions (Widodo et al., 2019, 2021). By providing information on the production, characterization, and bioactivity of these strains of EPSs, our study fills a critical gap in understanding the function of strain-specific EPSs, particularly from human-origin probiotic strains with health benefit effects. This study therefore provides evidence to support the probiotic claims of both strains, *L. casei* AP and *L. casei* AG, particularly when they are applied as cultures for the development of dairy-based functional foods.

## Materials and methods

### Bacterial strains

The *L. casei* strains AP and AG used in this study were deposited in the Food Nutrition and Culture Collection (FNCC; WDCM number 755) of the Inter University Centre Food and Nutrition, Universitas Gadjah Mada. The strains for *L. casei* AG and *L. casei* AP were FNCC 0500 and FNCC 0501, respectively. These bacteria were subcultured by inoculation on Man-Rogosa-Sharpe (MRS) agar (Merck, USA) supplemented with 0.5 g/L L-cysteine (Sigma‒Aldrich, USA) and incubated at 37 °C for 24 h under microaerobic conditions.

### Measurement of the bacterial growth curve

The active cultures of *L. casei* AP and AG were grown in 250 mL of liquid MRS medium supplemented with 0.5 g/L L-cysteine at 37 °C, and optical density (OD_600_) values were obtained at specific times during the 24 h of incubation. The number of viable cells and total plate count on MRS agar plates were also determined to calculate the number of colony-forming units (CFUs) per milliliter of sample.

### Extraction of crude EPSs and determination of the total carbohydrate content

EPS extraction was carried out according to a method previously reported by Chen et al. (2011), with modifications. The *L. casei* strains AP and AG were cultured in MRS broth at 37 °C for 28 h. The bacterial cells were separated by centrifugation (5,000 rpm for 20 min) at 4 °C. The supernatants were then supplemented with a solution of trichloroacetic acid (TCA) at a final concentration of 4% (m/v). The solutions were then centrifuged at 5,000 rpm for 20 min to eliminate the precipitated proteins. The supernatants were then precipitated with 99.9% cold ethanol (three volumes), stored overnight at 4 °C, and centrifuged at 5,000 rpm for 20 min at 4 °C. The precipitates obtained from centrifugation were dialyzed against deionized water using cellulose membrane dialysis tubing with a pore size of 12,000 Da (Merck, USA) for 48 h and then exchanged four times. The dialyzed solutions were then lyophilized for 36 h to obtain crude EPSs. The freeze-dried crude EPSs were weighed and analyzed for their carbohydrate content using the phenol‒sulfuric acid method (Dubois et al., 1956). Glucose at various concentrations ranging from 0 to 300 µg/mL was used as a standard.

### Structural characterization of crude EPSs

#### Fourier transform infrared (FTIR) analysis

The functional groups of EPSs isolated from the *L. casei* strains AP and AG were examined by FTIR spectroscopy following the methods described by Li et al. (2022). Commercial xanthan gum (Fufeng Meihua Deosen, Shandong, China) was used as a standard for EPSs. Two milligrams of each lyophilized EPS sample was mixed with potassium bromide (KBr) at a ratio of 1:100. Afterward, the compressed disk (13 mm) was scanned with FTIR (IRPrestige-21 SHIMADZU) in the wavelength range of 4000–400 cm^−1^.

#### Monosaccharide composition analysis

The monosaccharide composition of each EPS was determined by high-performance liquid chromatography (HPLC) as described previously (Vinothini et al., 2019). Briefly, 5 mg of EPS was hydrolyzed with 4 M trifluoroacetic acid (TFA), incubated at 100 °C for 2 h and then filtered through a 0.45 μm syringe filter. The excess TFA was removed by evaporation. The hydrolyzed EPS samples were injected into an HPLC system (Shimadzu LC-10/20, Japan) equipped with a Shim-pack SCR-101H column (7.9 mm × 30 cm) and detected using a refractive index detector. The temperature of the column was adjusted to 30 °C, and the wavelength of the detector was set to 210 nm. The mobile phase consisted of 100 mM perchloric acid, and the samples were eluted at a flow rate of 0.6 mL/min. Four target monosaccharides (glucose, galactose, xylose, and ribose) were chosen as internal standards. The concentration ranges of the monosaccharide standards used for retention time calibration were 0.25, 0.5, 1, 1.5, 2, and 2.5 mg/L.

#### Scanning electron microscopy (SEM) with energy-dispersive X-ray spectroscopy (EDX)

The morphology of each EPS sample was determined by scanning electron microscopy (SEM) using the protocol of Andrew and Jayaraman (2022) with modifications. Five-milligram samples of the EPSs from *L. casei* AP and *L. casei* AG were fixed on the stubs by coating them with a thin layer of gold under high vacuum conditions. The samples were subsequently examined using a scanning electron microscope (Axia ChemiSEM, Thermo Fisher, USA) at 2000 × magnification. Energy-dispersive X-ray spectroscopy (EDX) was used to analyze the elemental composition of the EPS samples.

### Immunomodulatory assay

#### Cell culture

The murine macrophage line RAW 264.7 was provided by the Parasitology Laboratory, Faculty of Medicine Public Health and Nursing, Universitas Gadjah Mada. RAW 264.7 cells were cultured in Dulbecco’s modified Eagle’s medium (DMEM; Sigma‒Aldrich, USA) supplemented with 10% fetal bovine serum (Gibco) at 37 °C in an incubator with a 5% CO_2_ atmosphere.

#### Viability assay

RAW 264.7 cells (1 × 10^4^ cells/well) were cultured in 96-well plates. After 24 h, the cells were further incubated for an additional 24 h with varying concentrations between 50 and 800 µg/mL EPS. The control group included cells without EPS treatment and was set to 100% cell viability. Subsequently, 100 µL of 3-(4,5-dimethylthiazolyl-2)-2,5-diphenyltetrazolium bromide (MTT; Sigma Aldrich, USA) solution was added to each well, and the mixture was incubated at 37 °C for 4 h. As a control, the formazan was dissolved in 100 µL of sodium dodecyl sulfate (SDS; Merck, USA), and the absorbance was measured at 595 nm. The viability of the RAW 264.7 cells was assessed using the following equation:

$${\text{Cell viability }}\left( \% \right) = {\text{Abs}}_{{{\text{sample}}}} {\text{/Abs}}_{{{\text{control}}}} \times {1}00$$

#### Macrophage phagocytosis

The phagocytic activity of RAW 264.7 cells was determined using a neutral red uptake assay following the method described by Qu et al. (2022) with modifications. RAW 264.7 cells (1 × 10^4^ cells/well) were incubated in a 96-well plate for 24 h and then treated with various concentrations of EPSs (50, 100, 200, 400 and 800 µg/mL) for another 24 h and with 1 µg/mL lipopolysaccharide (LPS) (Sigma‒Aldrich, USA) as a positive control. After the supernatant was discarded, 100 µL of 0.075% (v/v) neutral red solution (Sigma‒Aldrich, USA) was added to each well, and the mixture was incubated at 37 °C for 1 h. The excess supernatant was then removed, and 100 µL of the cell lysate was added. After the cells were incubated for two hours at 25 °C, the absorbance at 540 nm was measured. The phagocytosis index was calculated using the following equation:

$${\text{Phagocytosis index}} = {\text{Abs}}_{{{\text{sample}}}} /{\text{Abs}}_{{{\text{control}}}}$$

#### Nitric oxide (NO) production

RAW 264.7 cells (5 × 10^5^ cells/well) were cultured in a 24-well plate for 24 h. The cells were then incubated with various concentrations of EPSs (50, 100, 200, 400, and 800 µg/mL) or 1 µg/mL lipopolysaccharide (LPS) (as a positive control) for another 24 h. Finally, the concentration of NO in the supernatant was determined using the Griess method (Sun et al., 2003). Equal volumes of sulfanilamide (1% in 5% phosphoric acid) and n-naphthyl-ethylenediamine (0.1% in water) were mixed to prepare the Griess reagent. A standard curve was generated using various concentrations of NaNO_2_ (0–100 µM) to quantify nitrite in the samples. The absorbance was measured at 540 nm using a microplate reader.

### Statistical analysis

All the experiments were carried out in triplicate, and the data are presented as the means ± standard deviations (SDs). Statistical analyses were performed using GraphPad Prism 10 software. One-way analysis of variance (ANOVA) was used to compare data from more than two treatment groups. This analysis was followed by Tukey’s post hoc test to determine significant differences between groups. A p value < 0.05 indicated statistical significance in all analyses.

## Results and discussion

### Bacterial growth

**Fig. 1:**
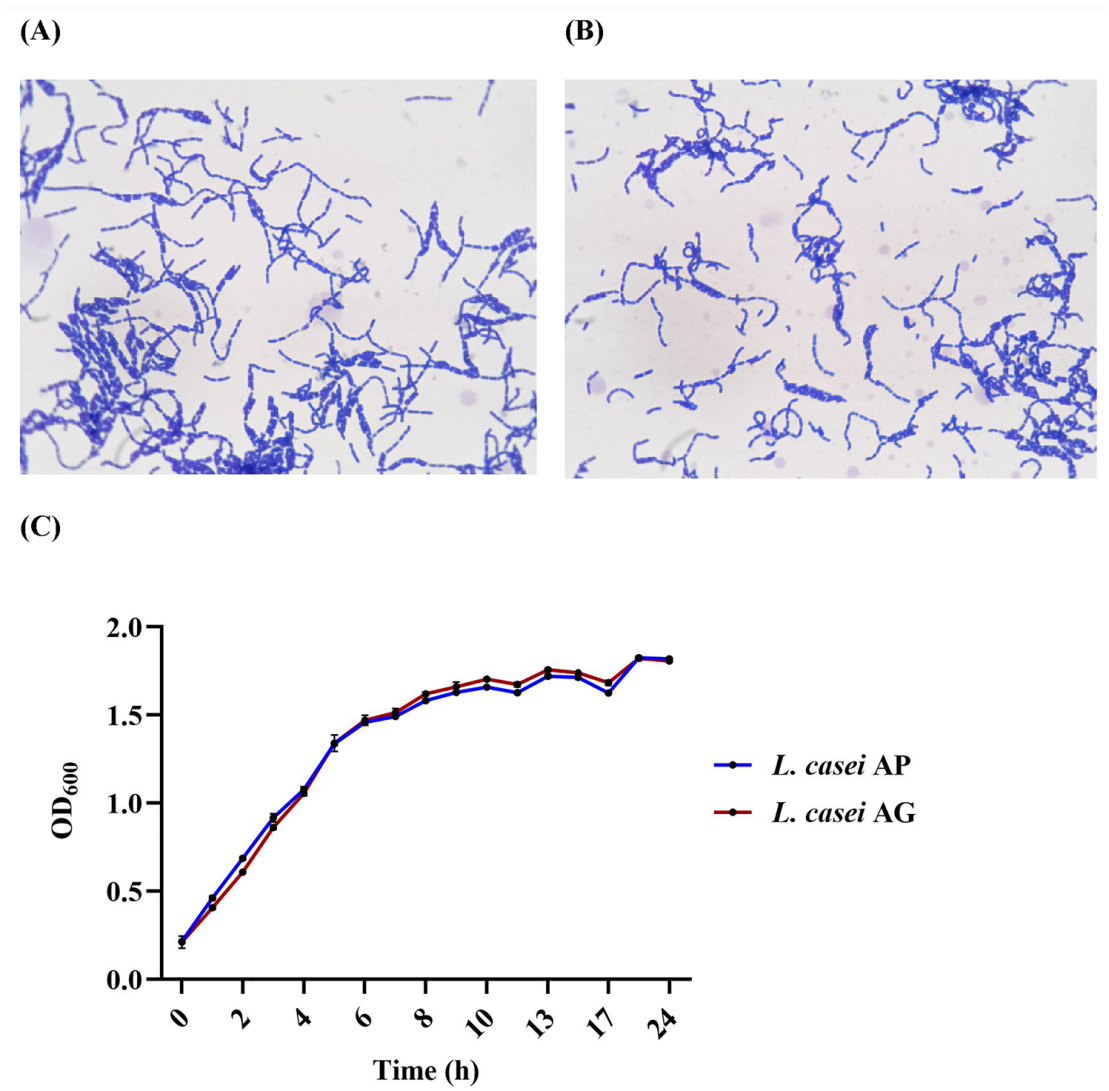
Morphologies of *L. casei* AP (**A**) and *L. casei* AG (**B**) under a phase-contrast microscope (1000 × magnification), with purple staining noted. Growth curves of *L. casei* AP and *L. casei* AG in MRS broth (**C**)

*Lacticaseibacillus casei* strains AP and AG were confirmed by Gram staining, and their cell morphologies were observed by phase contrast microscopy at 1000 × magnification (Fig. 1A and B). The rod-shaped bacterial cells are shown in purple in Fig. 1A and B, confirming that the *L. casei* strains AP and AG were gram-positive. Lactic acid bacteria are commonly known as heterogeneous groups of gram-positive bacteria. The growth stages of *L. casei* AP and AG in a batch culture were monitored for 24 h (Fig. 1C). As shown in Fig. 1C, the mid-logarithmic phases of the *L. casei* AP and *L. casei* AG cultures were observed at 4 h, and both bacterial cultures reached the stationary phase after 17 h of incubation. The total viable counts of *L. casei* AP and *L. casei* AG after 4 h of incubation were 7.0 × 10^8^ and 3.4 × 10^9^ CFU/mL, respectively. Moreover, the total numbers of viable *L. casei* AP and *L. casei* AG cells at 17 h were 4.3 × 10^9^ and 6.2 × 10^9^ CFU/mL, respectively. Monitoring the growth of *L. casei* AP and *L. casei* AG is essential because bacterial EPSs are synthesized in response to extreme environmental conditions such as nutritional depletion or after a stationary phase of growth is reached. Understanding the growth dynamics of bacterial strains also provides critical insights into the conditions and timing that maximize EPS yield for further research.

### Extraction and structural characterization of crude EPSs

With respect to EPS isolation, the growth of *L. casei* AP and *L. casei* AG was stopped after 28 h. Compared with *L. casei* AP (48 mg/L), *L. casei* AG yielded more EPSs (62 mg/L), with a slight difference in carbohydrate content. The carbohydrate contents in the EPS produced by *L. casei* AP and *L. casei* AG were 79.40 and 81.17%, respectively. Previous studies have reported obvious differences in the ability of LAB strains to produce EPSs, with concentrations ranging from 25 to 600 mg/L (Ruas-Madiedo, 2014). The weight of the EPS produced by the *L. casei* strains in this study was within a reasonable range. These findings were also in agreement with those of a previous study by Maajid et al. (2022). In this recent study, *L. casei* AP and *L. casei* AG were grown on standard MRS broth medium with 20 g/L glucose in its original formulation. Most LAB strains had EPS yields of less than 1 g/L under nonoptimized conditions (Badel et al., 2011). Carbon and nitrogen sources in the cultivation media affect EPS production. When glucose is used as a carbon source, *Lactobacillus delbrueckii* produces 175 mg/L EPS; on the other hand, a significantly lower production level of 69 mg/L EPS is obtained when it is grown on fructose as a carbon source (Yuksekdag and Aslim, 2008). Several other studies reported EPS production in other strains of lactic acid bacteria grown on skim milk lactose, e.g., 750 mg/L by *Lactobacillus casei* WXD030 (Xiu et al., 2020) and 1950 mg/L by *Streptococcus thermophilus* CC30 (Kanamarlapudi and Muddada, 2017). The yields of EPSs produced by *L. casei* AP and *L. casei* AG in this study were comparable to those produced by *Lactobacillus delbrueckii* when the bacteria were grown on fructose but lower than those produced by *Lactobacillus casei* WXD030 and *Streptococcus thermophilus* CC30 in the abovementioned studies. In this study, EPS production was low, possibly because of the preference for a carbon source. Some LAB strains produce more EPS when they are grown on disaccharides or complex sugars rather than on glucose (Fuso et al., 2023). Different carbon sources could be used in the cultivation media to further optimize EPS production by these strains. However, the optimization of EPS production is beyond the scope of this study, as our focus is on the physicochemical characterization and immunomodulatory effects of EPSs produced by these probiotic strains. Importantly, although the EPS yields obtained in this study (48–62 mg/L) were less than those reported for some LAB strains (> 500 mg/L), this work contributes to the field by investigating EPSs produced by novel indigenous strains isolated from the feces of Indonesian infants, which remain understudied in LAB research.

**Fig. 2:**
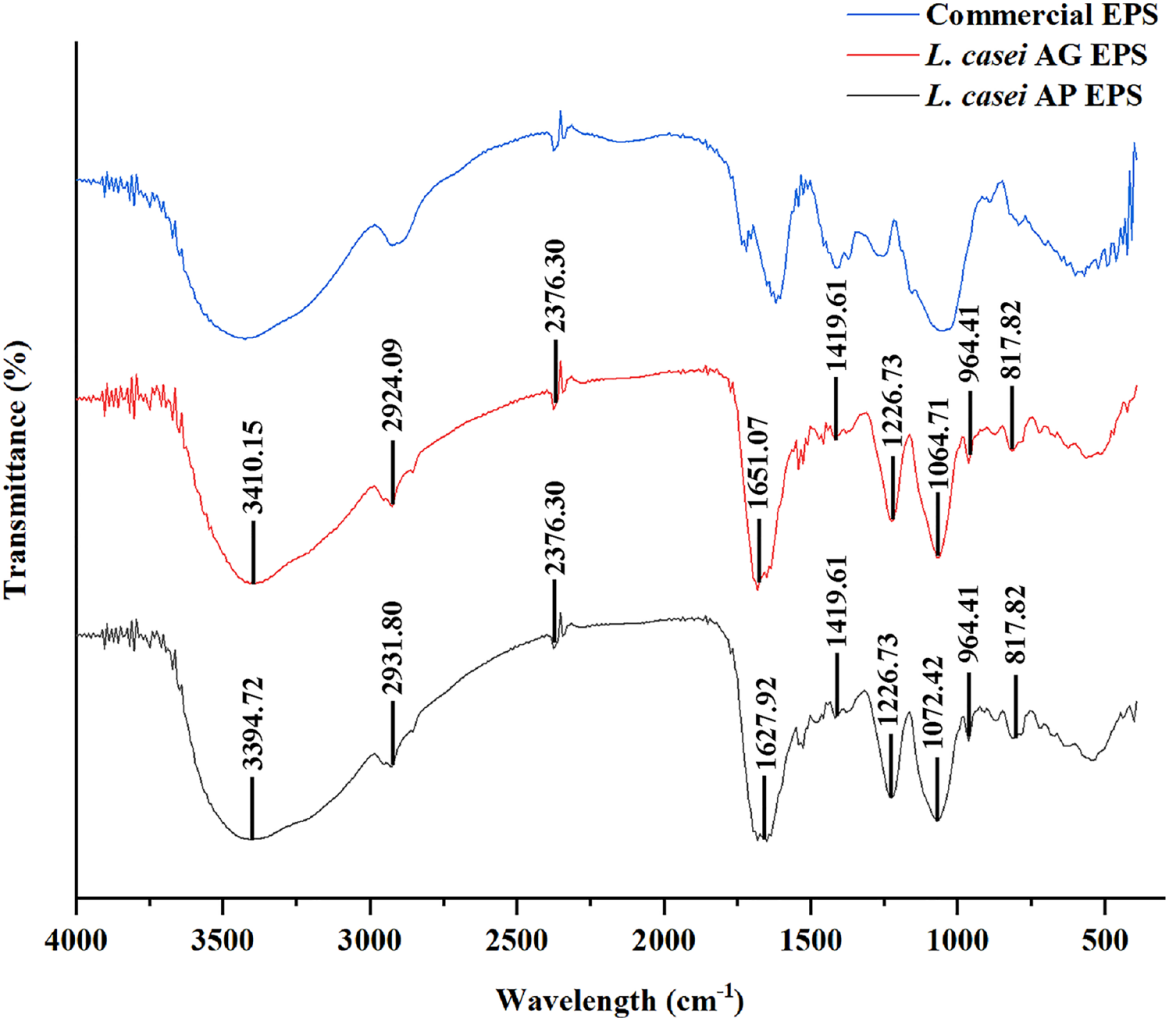
Fourier transform infrared (FTIR) spectra of the EPSs isolated from *L. casei* AP and *L. casei* AG in the range of 400–4000 cm^−1^. Xanthan gum (Fufeng Meihua Deosen, Shandong, China) was the commercial EPS used in this analysis

The functional groups of the EPSs were characterized using FTIR spectroscopy, and the FTIR spectra are shown in Fig. 2. The FTIR spectra of EPSs isolated from *L. casei* AP and *L. casei* AG were similar to those of commercially available EPSs (xanthan gum), suggesting that the functional groups of the isolated EPSs were similar to those of commercial samples. The wavelengths of the FTIR bands for each functional group are presented in Table 1, which shows various wavelengths of infrared radiation, suggesting the presence of various chemical bonds within the EPS samples. The spectra of the EPSs from *L. casei* AP and *L. casei* AG showed peaks at 3394.72 and 3410.15 cm^−1^, respectively, which indicated the presence of hydroxyl groups. The absorption of infrared radiation in the 2931.80 and 2924.09 cm^−1^ regions was related to the asymmetric C–H stretching vibration of the methylene (CH_2_) group. This CH_2_ group represents the presence of organic molecules in the EPS samples. The absorption peak at 2376.30 cm^−1^ was attributed to the N–H stretching band of the amide group, and the peaks at 1627.92 and 1651.07 cm^−1^ were attributed to the C=O stretching vibration of the N-acetyl group (Sardari et al., 2017). There was an absorption peak at 1419.61 cm^−1^, which likely indicated the presence of an ester or ether group (Wang et al., 2018). The absorption peak at 1240–11190 cm^−1^ indicated the presence of an aromatic phosphate group (P–O–C stretching). The absorption peak between 1000 and 1200 cm^−1^ was related to the vibration of the pyranose sugar ring. The sharp absorption peaks at 1072.42 and 1064.71 cm^−1^ indicated the presence of α-glycosidic bonds (Das and Goyal, 2013). Additionally, the absorption peak at 817.82 cm^−1^ confirmed the α-configuration of the EPS (Tian et al., 2021).

**Table 1:** Wavelength data from the FTIR spectra of the EPSs from *L. casei* AP and AG

Wavelength (cm^−1^)		Functional groups
EPS AP	EPS AG	
3394.72	3410.15	O–H *stretching*
2931.80	2924.09	C–H *stretching*
2376.30	2376.30	N–H *stretching*
1627.92	1651.07	C = O *stretching*
1419.61	1419.61	Ester or ether groups
1226.73	1226.73	Aromatic phosphate (P–O–C) stretch
1072.42	1064.71	α-(1 $\to$ 6) glycosidic bond
964.41	964.41	Aromatic ring C–H
817.82	817.82	α-configuration of EPSs

### Monosaccharide composition of the EPSs

**Fig. 3:**
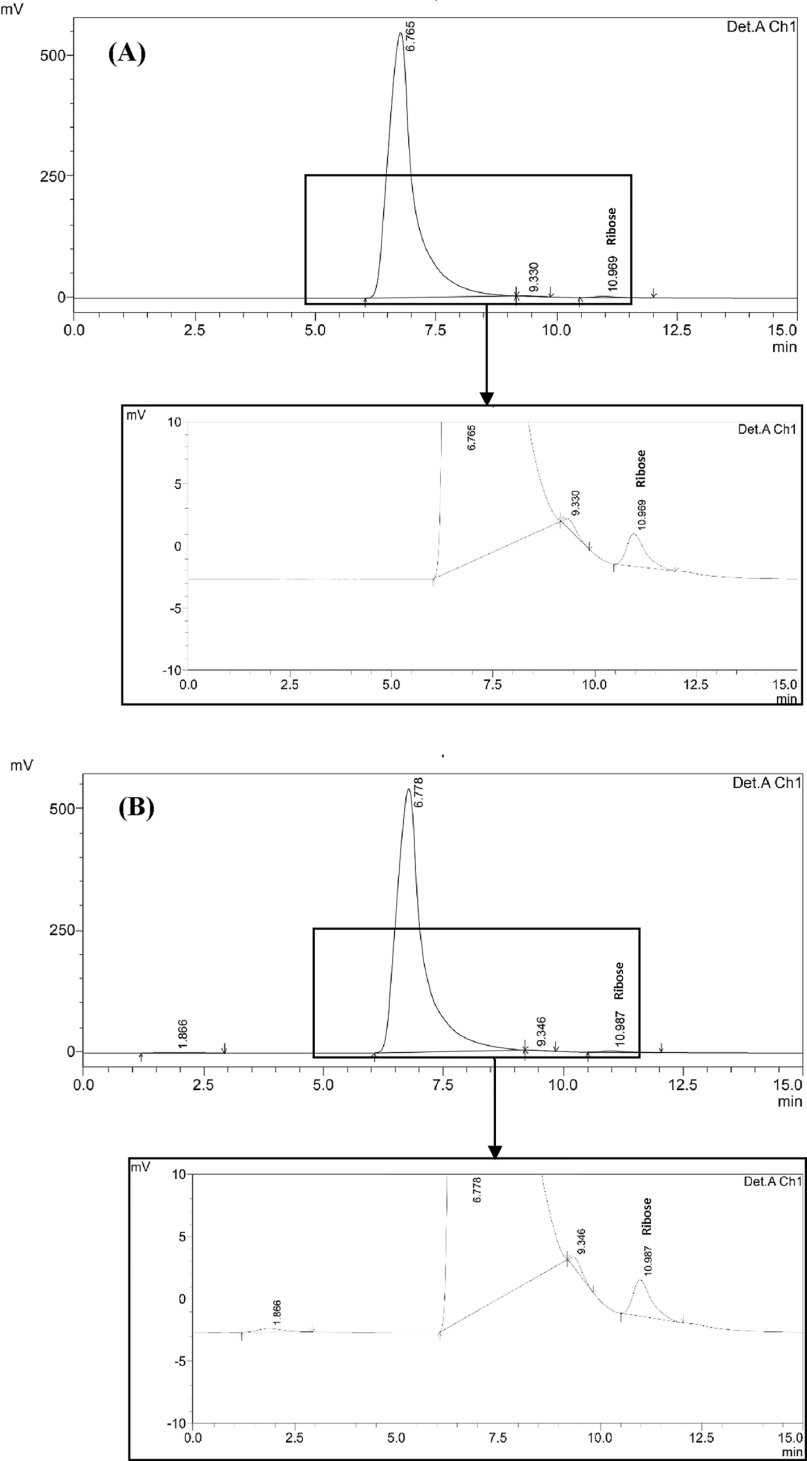

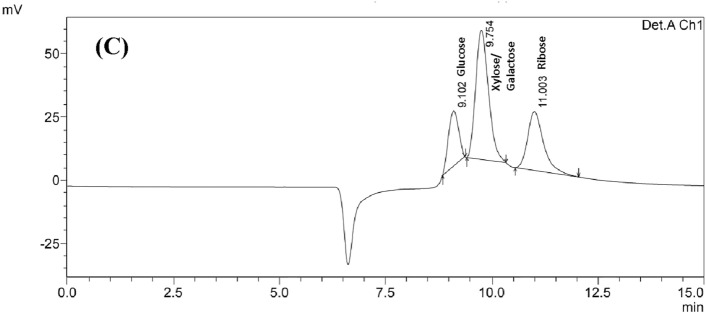
HPLC chromatograms of the EPS samples and the standard monosaccharides from *L. casei* AP (**A**), *L. casei* AG (**B**), and the monosaccharide standards (glucose, xylose, galactose, and ribose) (**C**). Chromatograms of EPS samples from *L. casei* AP and AG revealed the presence of the monosaccharides ribose and glucose, while xylose and galactose were not detected

The monosaccharide compositions of the EPSs isolated from *L. casei* AP and AG were analyzed by HPLC. Three peaks were observed at retention times of 6.7, 9.3, and 10.9 min (Fig. 3). The peak with a retention time of 10.9 min was attributed to ribose. The concentrations of ribose in the EPSs from both *L. casei* AP and *L. casei* AG were 0.10 and 0.13 mg/L, respectively. Moreover, the peak at 9.3 min detected for the EPSs from both *L. casei* AP and *L. casei* AG was close to the peak at 9.1 min attributed to glucose in the internal standards. The peak at a retention time of 9.3 min likely indicates the presence of glucose in the EPSs. There is a significant peak in the chromatograms presented in Fig. 3A and B at a retention time of approximately 6.7 min. However, since the peak at 6.7 min was not detected in the chromatogram of the monosaccharide standards (Fig. 3C), the compound responsible for this peak could not be identified. To accurately identify this peak, complementary analytical techniques with more sensitive compound detection capabilities, such as mass spectrometry or NMR spectroscopy, are needed. Moreover, expanding the set of monosaccharide standards used in HPLC analysis would provide accurate profiling of the monosaccharide composition in EPSs. Alternatively, the library built from synthesized standards proposed by Galermo et al. (2019) could be applied to identify missing compounds on the basis of the retention time library. These findings differ from the observations in previous studies, in which they reported the presence of glucose, arabinose, and mannose (Kavita et al., 2014); glucose, mannose, and galactose (Xiao et al., 2021); and glucose and galactose (Kanamarlapudi and Muddada, 2017) as the main monosaccharides of EPSs produced by various species of lactic acid bacteria. However, in some cases, ribose has been detected as a component of the EPS chain produced by lactic acid bacteria (LAB) (Pradeepa et al., 2016). Different bacterial strains, media and fermentation conditions likely contribute to the monosaccharide compositions of the EPSs produced by lactic acid bacteria.

### Microstructural and elemental analysis of the EPSs

**Fig. 4:**
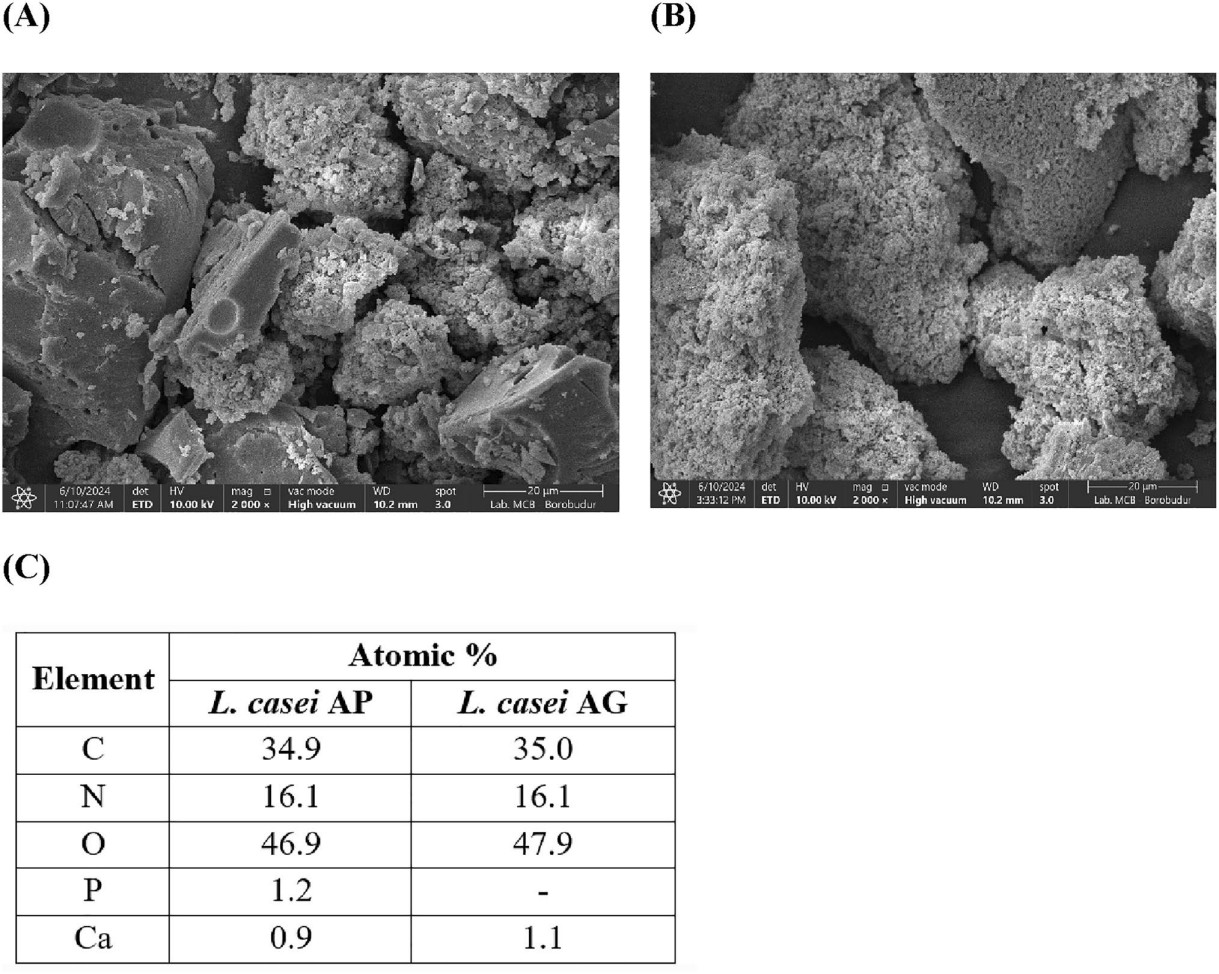
SEM images of the EPSs from *L. casei* AP (**A**) and *L. casei* AG (**B**) at 2000 × magnification and their elemental compositions (**C**)

Exopolysaccharides (EPSs) from *L. casei* AP and *L. casei* AG show irregular lumps with coarse surfaces (Fig. 4A and B), which could be beneficial for the formation of branched and interconnected networks of polysaccharides (Liu et al., 2018). These results were also similar to those of EPSs obtained from *Streptococcus thermophilus* (Kanamarlapudi and Muddada, 2017). Different bacterial strains can produce different EPS structures. Chegini et al. (2024) described the compact structure and smooth surfaces of EPSs from *Enterococcus faecium* PCH.25.

Energy-dispersive X-ray (EDX) spectroscopy revealed that oxygen was the main element of the EPSs isolated from *L. casei* AP and *L. casei* AG, with atomic percentages of 46.9 and 47.9%, respectively (Fig. 4C). The high proportions (percentages; %) of oxygen and carbon indicate that the main constituents of the EPSs from *L. casei* AP and *L. casei* AG were carbohydrates (Sirin and Aslim, 2020). Nitrogen (N) is the third most abundant elemental component, accounting for 16.1% of both EPS samples, suggesting the presence of amino bonds inside the sugar residue chain. Moreover, the presence of calcium and phosphorus in EPS samples might be due to their binding to the carboxyl and hydroxyl groups of monosaccharides (Mandal et al., 2011). In this study, phosphorus was detected only in the EPSs of *L. casei* AP at a proportion of 1.2%. Zaghloul and Ibrahim (2022) reported a lower proportion of phosphorus (0.99%) in EPSs produced by *Lactiplantibacillus plantarum* EI6, while oxygen (55.44%) and carbon (40.06%) accounted for the greatest proportion, with little nitrogen (2.15%) and the presence of additional elements such as chloride (1.03%), calcium (0.19%), sodium (0.12%), and magnesium (0.02%). The elemental composition of EPSs plays a substantial role in determining the structural and functional traits of exopolysaccharides (Amer et al., 2021). The discovery of phosphate groups in EPSs produced by *L. casei* AP is a significant finding that contributes to a better understanding of the biochemical composition and potential bioactivity of these biopolymers. The presence of substituent groups such as phosphate moieties critically influences the biological functions of EPSs (Liu et al., 2025). EPSs containing phosphate exhibit proinflammatory properties and stimulate immune responses. The presence of phosphate groups can activate the immune system by triggering immune cells such as macrophages and lymphocytes (Saadat et al., 2019). Phosphate is a molecule that triggers the immunological response, as chemical dephosphorylation of EPSs weakens their stimulatory effect (Hidalgo-Cantabrana et al., 2012).

### Cytotoxicity of EPSs to RAW264.7 cells

**Fig. 5:**
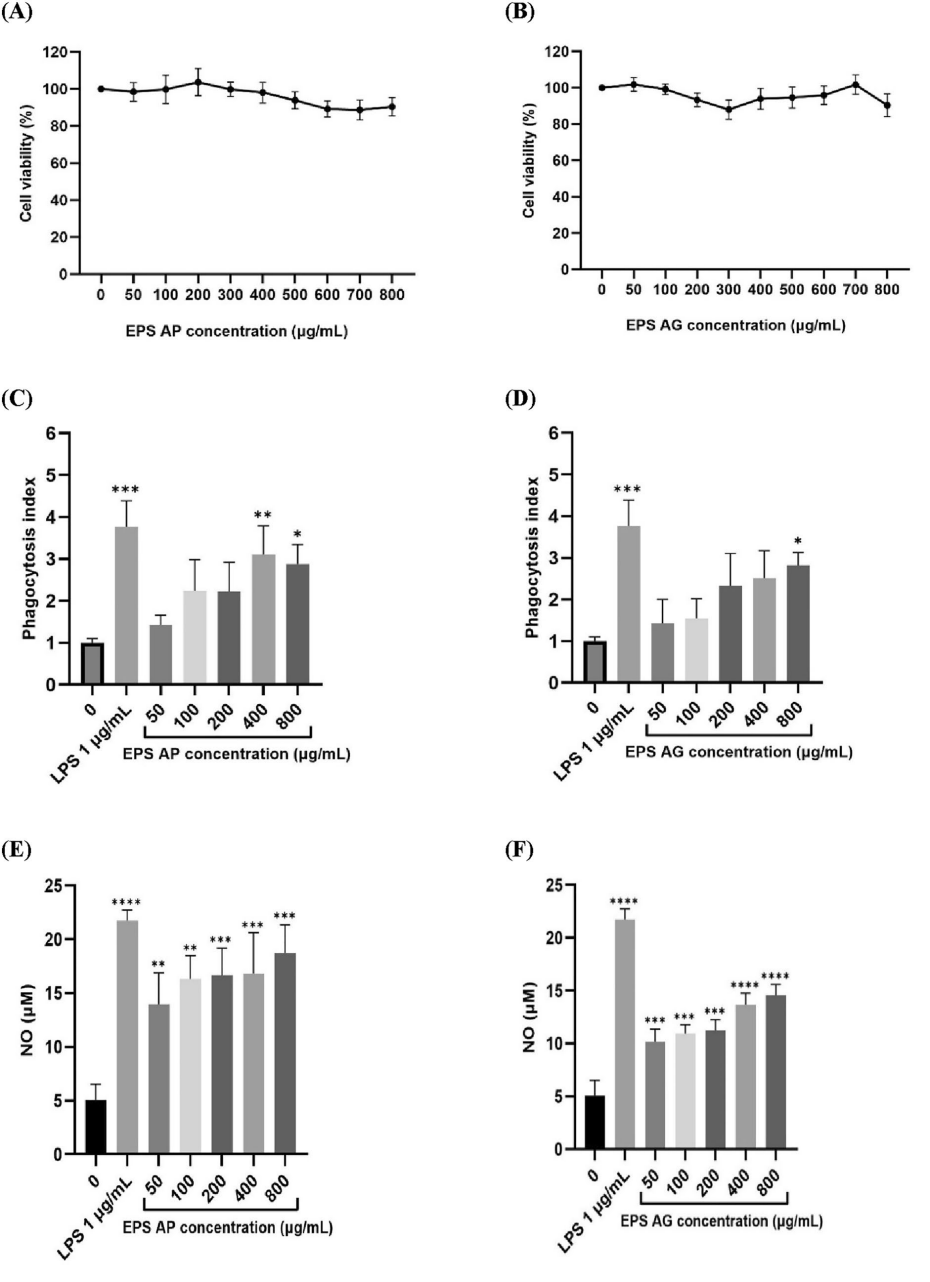
Effects of various concentrations (μg/mL) of the EPSs from *L. casei* AP and *L. casei* AG (**A** and **B**) on RAW 264.7 cell viability (%); the phagocytosis index of the EPSs from *L. casei* AP and *L. casei* AG (**C** and **D**); and the nitric oxide (NO) production levels after RAW 264.7 cells were incubated with varying concentrations of the EPSs from *L. casei* AP and *L. casei* AG (**E** and **F**). The data are expressed as the mean ± SD of 3 replicates with a total of 24 samples. The levels of significant differences compared with those of the control group are noted in the figures as follows: *(p < 0.05), **(p < 0.01), ***(p < 0.001), ****(p < 0.0001), # (p < 0.05), ## (p < 0.01), ### (p < 0.001), and #### (p < 0.0001). LPS at a concentration of 1 μg/mL was used as a positive control

The MTT assay is a colorimetric method used to measure metabolic activity within cells as an indicator of cell viability and is therefore an indirect method used to measure cytotoxicity. As the EPS concentration of *L. casei* AP increased, the cell viability initially increased at a concentration of 200 µg/mL but then began to decrease. The cell viability reached a maximum of 103.73% for the AP EPS and 101.84% for the AG EPS. The decrease in cell viability was still greater than 85%, although there was no significant difference compared with that in the control group. A cytotoxicity assay of the EPSs isolated from *L. casei* AP and *L. casei* AG revealed that the EPSs at concentrations ranging from 50,800 µg/mL were not toxic to RAW 264.7 cells (Fig. 5A and B) and could be used for subsequent experiments.

Exopolysaccharides (EPSs) are known for their immunomodulatory activity, as they can modulate the immune system through multiple mechanisms (Yang et al., 2023). RAW 264.7 cells are extensively used to investigate the immunological activity of EPSs, which trigger the innate immune response and inflammation by increasing phagocytosis, nitric oxide (NO) production, and cytokine secretion (Ren et al., 2017). The cytotoxic effects of EPSs on macrophages were measured by an MTT (3-(4,5-dimethylthiazol-2-yl)-2,5-diphenyltetrazolium bromide) assay. Our study revealed no toxicity of the isolated EPSs to RAW 264.7 cells at concentrations ranging from 50–800 µg/mL (Fig. 5A and B). Our findings agreed with those of a previous study of EPSs derived from *L. plantarum* JLK0142, which were not toxic to RAW 264.7 cells at concentrations ranging from 50 to 1000 μg/mL (Wang et al., 2018).

### Effect of EPSs on the phagocytic capacity of RAW264.7 cells

Phagocytosis is a fundamental process in the immune system, and macrophages are key players in this defensive mechanism. Phagocytosis is crucial for maintaining tissue homeostasis, fighting infections, and initiating further immune responses (Rosales and Uribe-Querol, 2017). Phagocytic ability may indicate the activity or functional capacity of macrophages. RAW 264.7 cells were treated with various concentrations of EPSs (50–800 µg/mL) to evaluate their effects on the phagocytosis index. Exopolysaccharides (EPSs) at concentrations ranging from 50 to 800 µg/mL resulted in a significantly higher phagocytosis index than that of the control group (without EPSs, i.e., 0 µg/mL EPS) (Fig. 5C and D) but had a lower phagocytosis index than that of the positive control group (treated with 1 µg/mL LPS). The EPS from *L. casei* AP increased the phagocytic activity to 310% at a concentration of 400 µg/mL, whereas the EPS from *L. casei* AG showed dose-dependent effects and reached the highest phagocytic activity of 282% at a concentration of 500 µg/mL. These findings suggested that EPSs isolated from *L. casei* AP and *L. casei* AG could activate RAW264.7 cells by promoting phagocytosis. The phagocytic capacity of the EPSs isolated from *L. casei* AP was greater than that of the EPSs isolated from *L. casei* AG. Other studies have demonstrated that EPSs produced by different species of lactic acid bacteria can increase the phagocytic capacity of macrophages. EPSs isolated from *L. kefiri* increased the phagocytic activity to 175% at a concentration of 500 µg/mL (Xiu et al., 2020). Although both strains belong to the same species, *L. casei* AP and *L. casei* AG exhibit different phagocytic capacities, and this difference might be related to differences in their structures and elemental compositions.

### Effects of EPSs on the NO production of RAW264.7 cells

As a major component of the host defense system, macrophages can modulate the immune system by generating various mediators and cytokines. Nitric oxide (NO) is an intracellular molecule that regulates a diverse range of physiological processes and plays a role in immune responses (Alderton et al., 2001). Nitric oxide production is commonly regarded as a signal of the inflammatory response. Thus, the level of NO production in this study was used to measure macrophage activation. As shown in Fig. 5E and F, the EPSs from *L. casei* AP and *L. casei* AG significantly promoted the production of NO after RAW 264.7 cells were incubated with varying concentrations of the EPSs from *L. casei* AP and *L. casei* AG. Furthermore, the amount of NO produced after the incubation of RAW 264.7 cells with EPSs from *L. casei* AP was greater than that with the EPSs from *L. casei* AG, especially at the highest EPS concentration of 800 µg/mL, in which the amounts of NO produced reached 18.730 and 14.603 µM, respectively.

Factors such as molecular weight, conformation, degree of branching, glycosidic connections and monosaccharide composition of EPSs affect their biological activity (Hidalgo-Cantabrana et al., 2012). Both *L. casei* strains in our study displayed efficient NO production over a wide range of EPS concentrations. Moreover, compared with that in the control group, NO secretion in the LPS group, which served as a positive control, significantly increased, reaching 21.75 μM (p < 0.05) (Fig. 5C). LPS was used as a positive control because of its ability to enhance immune responses and serve as a common model for inducing macrophage activation (McCall et al., 2020). The results demonstrated a dose-dependent increase (50–800 μg/mL) in NO generation in RAW264.7 cells stimulated by EPSs from *L. casei* AP and AG. The increase in NO secretion suggested that EPSs stimulate macrophage immunomodulatory activity. Exopolysaccharides produced by LAB modulate the immune response by inducing the secretion of proinflammatory mediators such as tumor necrosis factor-α (TNF-α), interleukin-6 (IL-6), interleukin-12 (IL-12), and inducible nitric oxide synthase (iNOS). Exopolysaccharides (EPSs) may improve their interaction with immune cell receptors, such as toll-like receptor 4 (TLR4) and Dectin-1, by adopting a triple helix conformation. This structural feature facilitates the activation of key signaling pathways, including NF-κB and MAPK, which in turn increases the production of various immune mediators (Gao et al., 2022). The observed difference in NO production might be attributed to the presence of phosphate groups in the EPSs from *L. casei* AP but their absence in the EPSs from *L. casei* AG. Studies have demonstrated that EPSs act as stimulators of immune cells when there are phosphate groups in their structures, making them negatively charged (Saadat et al., 2019). This is supported by findings in which the chemical removal of phosphate groups from EPSs significantly diminished their capacity to stimulate immune responses (Hidalgo-Cantabrana et al. (2012). While phosphate groups contribute to the immunostimulatory effects of EPSs, the overall immune response is a product of multiple structural and physicochemical factors. Another correlation was identified for high-molecular-weight EPSs, which function as immunosuppressive agents by attenuating the immune response (Hidalgo-Cantabrana et al., 2012; Ruas Madiedo, 2014). Additionally, the three-dimensional conformation and complexity of branched chains are crucial factors influencing their immunomodulatory effects (Ismail and Nampoothiri, 2013). Further research is necessary to elucidate the precise mechanisms underlying the role of EPSs as immunomodulators.

In conclusion, the EPSs isolated from the two probiotic strains *L. casei* AP and *L. casei* AG were physiochemically characterized in detail to determine their ability to modulate the response of immune cells. Although both strains belong to the same species, the isolated EPSs displayed differences in carbohydrate contents, structures and elemental compositions as well as the ability to induce immune responses. These EPSs were shown to increase phagocytic activity and promote the generation of nitric oxide in RAW 264.7 cells, which confirms that they possessed macrophage-activating activity. Details on the physicochemical characterization and immunomodulatory effects of the EPSs produced by these probiotic *L. casei* strains suggest that these strains are interesting candidates for the production of EPSs, which could be of great interest for applications in the pharmaceutical, food and dairy industries. Hence, further optimization to increase the production of EPSs using these *L. casei* strains is indeed desirable.
